# Rapid Energy Exchange between In Situ Formed Bromine Vacancies and CO_2_ Molecules Enhances CO_2_ Photoreduction

**DOI:** 10.34133/research.0244

**Published:** 2023-10-05

**Authors:** Qin Ren, Ye He, Hong Wang, Yanjuan Sun, Fan Dong

**Affiliations:** ^1^ Research Center for Environmental and Energy Catalysis, Institute of Fundamental and Frontier Sciences, University of Electronic Science and Technology of China, Chengdu 611731, China.; ^2^ School of Resources and Environment, University of Electronic Science and Technology of China, Chengdu 611731, China.

## Abstract

Photocatalytic reduction of CO_2_ into fuels provides a prospective tactic for regulating the global carbon balance utilizing renewable solar energy. However, CO_2_ molecules are difficult to activate and reduce due to the thermodynamic stability and chemical inertness. In this work, we develop a novel strategy to promote the adsorption and activation of CO_2_ molecules via the rapid energy exchange between the photoinduced Br vacancies and CO_2_ molecules. Combining in situ continuous wave-electron paramagnetic resonance (cw-EPR) and pulsed EPR technologies, we observe that the spin–spin relaxation time (T_2_) of BiOBr is decreased by 198 ns during the CO_2_ photoreduction reaction, which is further confirmed by the broadened EPR linewidth. This result reveals that there is an energy exchange interaction between in situ formed Br vacancies and CO_2_ molecules, which promotes the formation of high-energy CO_2_ molecules to facilitate the subsequent reduction reaction. In addition, theoretical calculations indicate that the bended CO_2_ adsorption configuration on the surface of BiOBr with Br vacancies caused the decrease of the lowest unoccupied molecular orbital of the CO_2_ molecule, which makes it easier for CO_2_ molecules to acquire electrons and get activated. In situ diffuse reflectance infrared Fourier transform spectroscopy further shows that the activated CO_2_ molecules are favorably converted to key intermediates of COOH*, resulting in a CO generation rate of 9.1 μmol g^−1^ h^−1^ and a selectivity of 100%. This study elucidates the underlying mechanism of CO_2_ activation at active sites and deepens the understanding of CO_2_ photoreduction reaction.

## Introduction

Economically and sustainably converting CO_2_ and H_2_O by solar energy and photocatalysts can realize the production of solar fuels and valuable chemicals in a more eco-friendly manner [1–4]. Unfortunately, the activation of the CO_2_ molecule is extremely difficult, because a CO_2_ molecule has 2 stable delocalized π_3_^4^ bonds with a high dissociation energy of 799 kJ mol^−1^ for C=O [5–7]. The photocatalytic CO_2_ reduction process is related to the active sites for the activation of the C=O bond, where the activation of the double bond is considered to be the rate-determining step for CO_2_ reduction. Of note, the addition of a single electron induces a bending of the molecular structure, thereby decreasing the energy barrier of CO_2_ activation [8–10]. Consequently, it is crucial to promote the activation of CO_2_ molecules by regulating the active sites with abundant localized electrons.

Recently, defect engineering is considered as a promising strategy to availably optimize the electronic structure, facilitating the adsorption and activation of CO_2_ molecules, thereby improving the inducing activity of CO_2_ molecules [11–13]. As reported previously, the surface defects with abundant localized electrons can serve as highly active sites for catalytic reactions, which involve electron transfer between the catalytic surface and reactant molecules [14–17]. However, the interactions between electrons and reactant molecules make the photophysical and photochemical processes difficult to understand and control owing to the lack of in situ characterization techniques [18–21]. Thus, it is highly advisable, though challenging, to develop more accurate and advanced in situ characterization techniques to track the dynamic process of electrons and advance the understanding of the underlying CO_2_ activation mechanism in the photocatalytic reduction process.

In this work, we utilize the tetragonal BiOBr (BOB) as a prototype catalyst to explore the interactions between CO_2_ on the ground state and the excited state on catalyst sites, which is the key to elucidate the in-depth reaction mechanism. Multiple (quasi) in situ characterizations were employed to investigate the influence of the dynamic electronic behavior with structure evolution on the photocatalytic CO_2_ activation process, including in situ electron paramagnetic resonance (EPR), in situ Raman, in situ x-ray photoelectron spectroscopy (XPS), and in situ diffuse reflectance infrared Fourier transform spectroscopy (DRIFTS). The spin centers of excited state on photoinduced Br vacancies (PI-BrVs) could transfer energy to CO_2_ molecules, generating the high-energy CO_2_ molecules that are more likely to acquire electrons and be activated. Theoretical calculations further verify that the reduction of the lowest unoccupied molecular orbital (LUMO) of CO_2_ molecules is decreased, which makes it easier to be adsorbed and activated, which is a key step for CO_2_ activation but has been largely overlooked. The rapid energy exchange between PI-BrVs and CO_2_ molecules enables a CO generation rate of 9.1 μmol g^−1^ h^−1^ and a product selectivity of 100%. Meanwhile, the conversion process of the activated CO_2_ molecules is further investigated and revealed by in situ DRIFTS. This work provides a new perspective into the activation mechanism of CO_2_ molecules and deepens the understanding of photocatalytic CO_2_ reduction reaction.

## Results

### The generation of in situ photoinduced Br vacancies

**Fig. 1.**
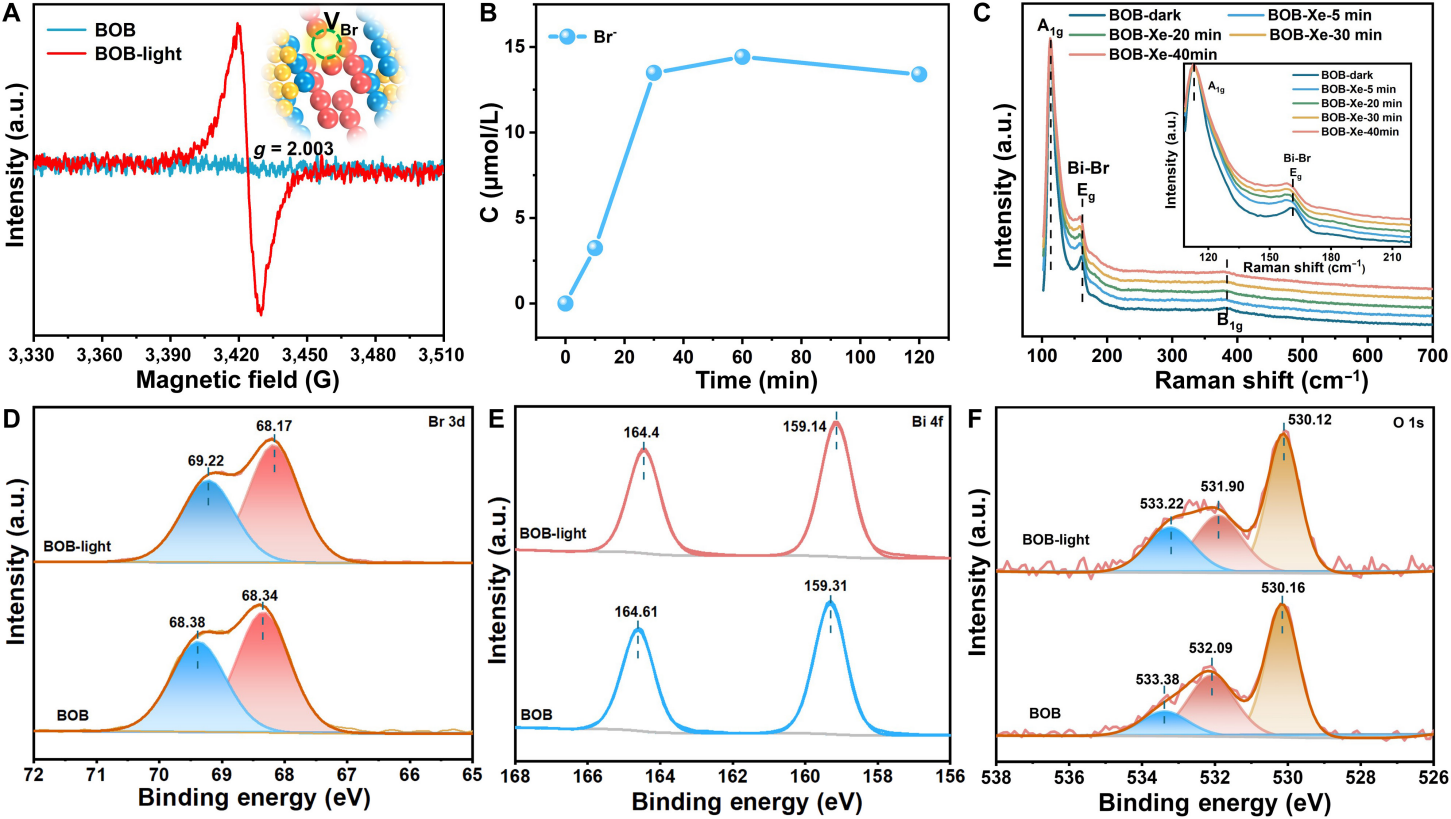
(A) EPR spectra of BOB sample before and after light irradiation. (B) The Br^−^ ion precipitation concentration with light exposure. (C) Raman spectra of BOB sample before and after light irradiation. (D) Br 3d of XPS spectra, (E) Bi 4f of XPS spectra, and (F) O 1s of XPS spectra.

Catalysts are inherently dynamic during a reaction since they could change their local structure for responding to the environment [22–25]. While the reconstruction of catalyst during the reaction is a recognized phenomenon, it can be disclosed via catalytic activity trends. Analyzing the state of reconstruction allows distinguishing real working catalytic sites, so as to design new catalysts [26–28]. In this work, the dynamic surface reconstruction of BOB samples during photocatalytic CO_2_ reduction and its influence on the catalytic reaction was studied. The x-ray diffraction pattern matches the tetragonal phase BOB (JCPDS 09-0393) in Fig. S1. The transmission electron microscopy (TEM) image showed 2D sheet-like morphology in Fig. S2A, while the high-resolution transmission electron microscopy (HRTEM) image in Fig. S2B revealed the high orientation along the [010] projection of tetragonal phase BOB, which has an open channel structure with the surface atomic alternation of [Bi_2_O_2_]^2+^ and Br^−^ on the facet. More importantly, the *g* value of 2.003 in the EPR spectrum consists of a single line of typical Lorentzian shape, confirming the existence of vacancies after Xe light irradiation (Fig. 1A) [29]. To investigate the types of vacancy generated on the BOB surface after light irradiation, the precipitation of Br^−^ on the surface of BOB samples with different illumination times was determined by ion chromatography (IC). With the prolonged illumination time, the concentration of Br^−^ gradually increased and reached equilibrium after 30 min in Fig. 1B. Therefore, we speculate that Br vacancies are generated on the BOB surface after irradiation. In addition, according to the following formula based on Hooke’s law: $=\frac{1}{2\pi c}\sqrt{\frac{k}{\mu }}$, where *ω* is Raman shift (cm^−1^), *c* is the velocity of light, and *μ* is the effective mass. The Raman shift of the Bi–Br bond (e.g., 162 cm^−1^) reduces after illumination, attributed to the decreased force constant (*k*) of the Bi–Br bond due to the introduced Br vacancy [30–32]. This result further confirms the generation of Br vacancies during the light irradiation in Fig. 1C.

For surface structure analysis, quasi-in situ XPS was conducted. In the high-resolution XPS Br 3d spectra of the BOB sample in Fig. 1D, there are 2 peaks at 68.38 (Br 3d_3/2_) and 68.34 eV (Br 3d_5/2_) [33]. After light irradiation of the surface of the BOB sample, the Br 3d shifted to lower binding energy, demonstrating the local chemical environment change resulting from the generation of PI-BrVs. The Bi 4f XPS spectra of BOB exhibit the characteristic spin-orbit doublet splitting center at 159.31 and 164.61 eV in Fig. 1E [34]. In contrast, the Bi 4f spectrum of BOB-light shows a shift toward lower binding energy relative to the BOB, indicating the potential breakage of Bi–Br bonds for PI-BrVs formation. Besides, 3 oxygen-containing species in the O 1s XPS spectra can be assigned to the lattice oxygen atoms near the surface (about 530.14 eV), surface oxygen (about 532.05), and adsorbed oxygen on the sample surface (about 533.32) in Fig. 1F [35,36]. After light irradiation, the O 1s XPS spectra have a slight shift toward lower binding energy, which is attributed to the introduction of Br vacancies. Consequently, Br vacancies are generated on the surface of BOB during light irradiation.

### The energy exchange mechanism between CO_2_ molecules and Br vacancies

**Fig. 2.**
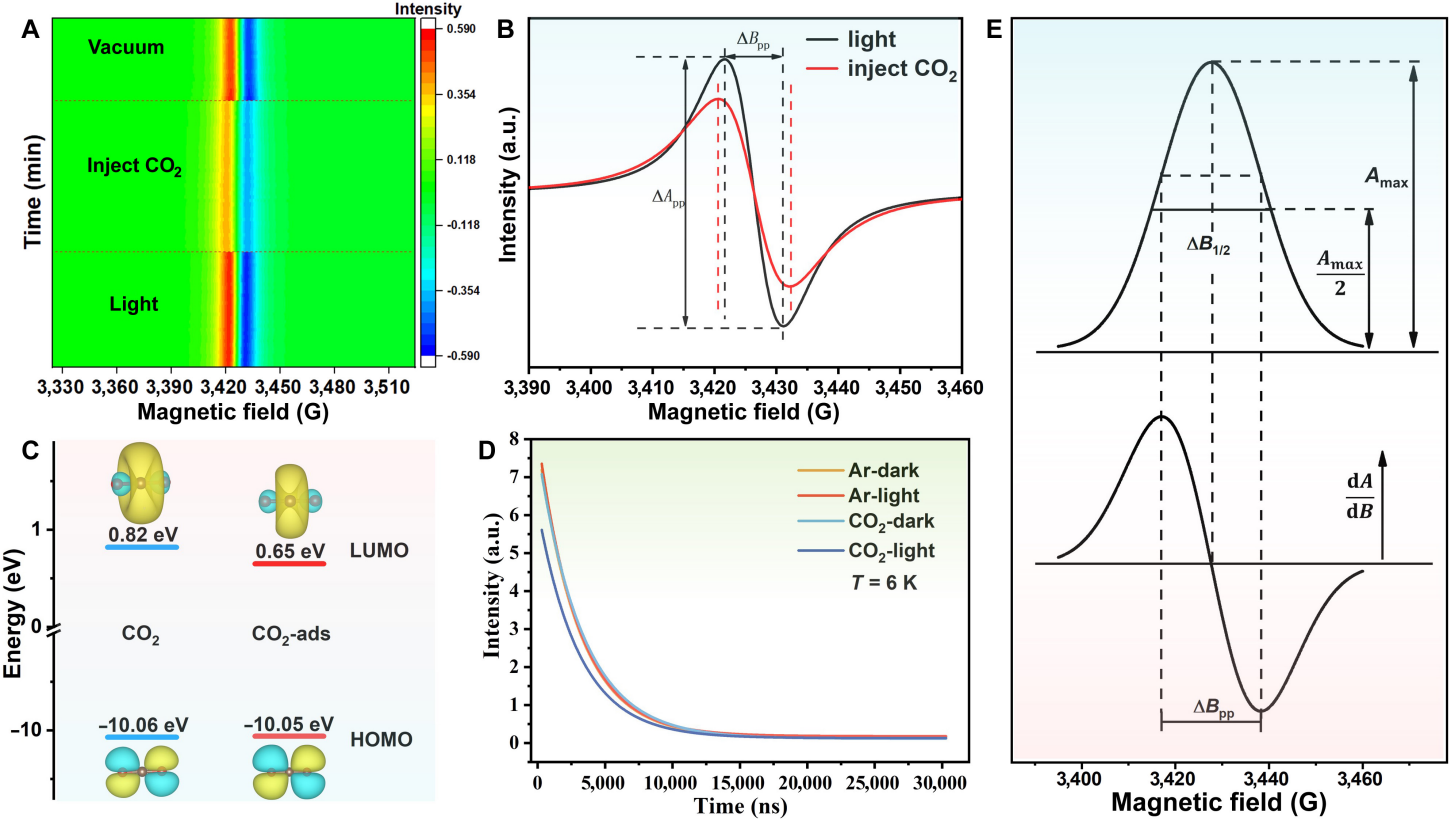
(A) The visualization of in situ EPR spectra on BOB sample under light irradiation, in CO_2_ atmosphere, and in a vacuum, respectively. (B) EPR spectra before and after CO_2_ injection. (C) Highest occupied molecular orbital (HOMO) and LUMO energy of CO_2_ molecules before and after adsorption. (D) Spin–spin relaxation time (T_2_) was measured by pulse EPR under different conditions. (E) The absorption of EPR (A) and its first derivative (dA/dB) as a function of magnetic field strength (B).

To investigate the electronic interactions between the PI-BrVs and CO_2_ molecules, in situ EPR experiments were conducted under a CO_2_ atmosphere. The in situ EPR visualization map of the BOB sample is shown in Fig. 2A. The BOB sample was illuminated for 2 h in a vacuum by a 300-W xenon lamp with an AM 1.5 G filter. The light-exposed BOB sample was transferred to a home-built in situ EPR catalytic reactor (Fig. S3). This in situ EPR device can continuously record the spectra under light irradiation, in gas atmosphere, or in a vacuum condition. Then, we recorded the in situ EPR spectra of the light-exposed BOB sample in a vacuum. Under light irradiation for 40 min, the EPR spectra have barely changed, indicating that the surface PI-BrVs have reached saturation after light irradiation of 2 h. Subsequently, EPR spectra intensity reduced with the injection of CO_2_ molecules, demonstrating that electrons transfer from the surface of BOB to CO_2_ molecules, which is a crucial step for CO_2_ molecule activation. Furthermore, theoretical calculation suggests that the adsorbed CO_2_ molecules on the BOB sample with Br vacancy have more obvious bending of the molecular structure than the BOB sample (Fig. S4A and B). The non-linear CO_2_ molecules are more unstable than linear CO_2_ molecules and exhibit a higher reactivity in CO_2_ photoreduction. The energy level of the LUMO is lowered with the O=C=O bond angle reducing, thus decreasing the energy barrier for CO_2_ activation (Fig. 2C). Therefore, the bent CO_2_ molecules could be easily activated via electron transfer from Br vacancy to the unpopulated antibonding 1π_u_* orbital of a CO_2_ molecule (Fig. S5). Of note, after the injection of CO_2_ molecules, the EPR signal linewidth is wider as shown in Fig. 2B. According to Heisenberg’s uncertainty principle, ∆*E* ∙ ∆*t* ≈ ℏ can be described by the following equation:

$$∆\nu ∙∆t=\gamma_{e}∆B∙∆t\approx 1/2\pi$$ (1)

Of note, Δ*ν*(=*γ*_e_∆*B*) (Hz) or ∆*B* (mT) means the linewidth of the EPR signal, and ∆*t* (s) stands for the lifetime of the spin state. A long- (or short-) lifetime state would produce a narrow (or wide) EPR signal.

The spin state lifetime (∆*t*) for *α* (*Ms* =  + 1/2; spin up) or *β* (*Ms* =  −1/2; spin down) is decided by relaxation times T_1e_ and T_2e_:

$$1/∆t\approx \left(1/T_{1e}\right)+\left(1/T_{2e}\right)$$ (2)

T_1e_ stands for the spin-lattice relaxation (SLR) time and T_2e_ stands for the spin–spin relaxation (SSR) time. SLR controls the energy exchange between the spin ensemble and the environment (lattice), while SSR includes the interactions within the ensemble. In general, the relationships T_1e_ ≫ T_2e_ and 1/T_1e_ ≪ 1/T_2e_ generally hold, leading to

$$1/∆t\approx 1/T_{2e}$$ (3)

Therefore, following the uncertainty principle, the linewidth is

$$∆\nu =\gamma_{e}∆B\propto 1/∆t\approx 1/T_{2e}$$ (4)

The EPR signal is typically expressed as absorption of the first derivative of *A* with respect to *B* as a function of *B*, d*A*/d*B* (Fig. 2E). The shape of *A* could be a Gaussian curve or a Lorentzian curve or be a suitable mixture of the two, where T_2e_ is multiplied by a function of T^2^_1e_, with T^2^_2e_ either in the exponent (Gaussian) or in the denominator (Lorentzian). The eigen values are the maximum *A*_max_ of *A*, the peak width of its half-height (*A*_max_/2) is **∆***B*_1/2_, and the peak-to-peak distance of the distance **∆***B_pp_* of the derivative curve d*A*/d*B* [37,38]. Therefore, the SSR time T_2e_ decreases as linewidth broadens. Advanced in situ continuous wave-electron paramagnetic resonance (cw-EPR) and pulsed EPR technologies were applied to investigate the interactions between in situ generation Br vacancies and CO_2_ molecules during the photoreduction process and reveal the mechanistic insights into the photocatalytic reduction of CO_2_, which provides technical support for the study of photocatalytic CO_2_ reduction reaction from a fresh point.

**Fig. 3.**
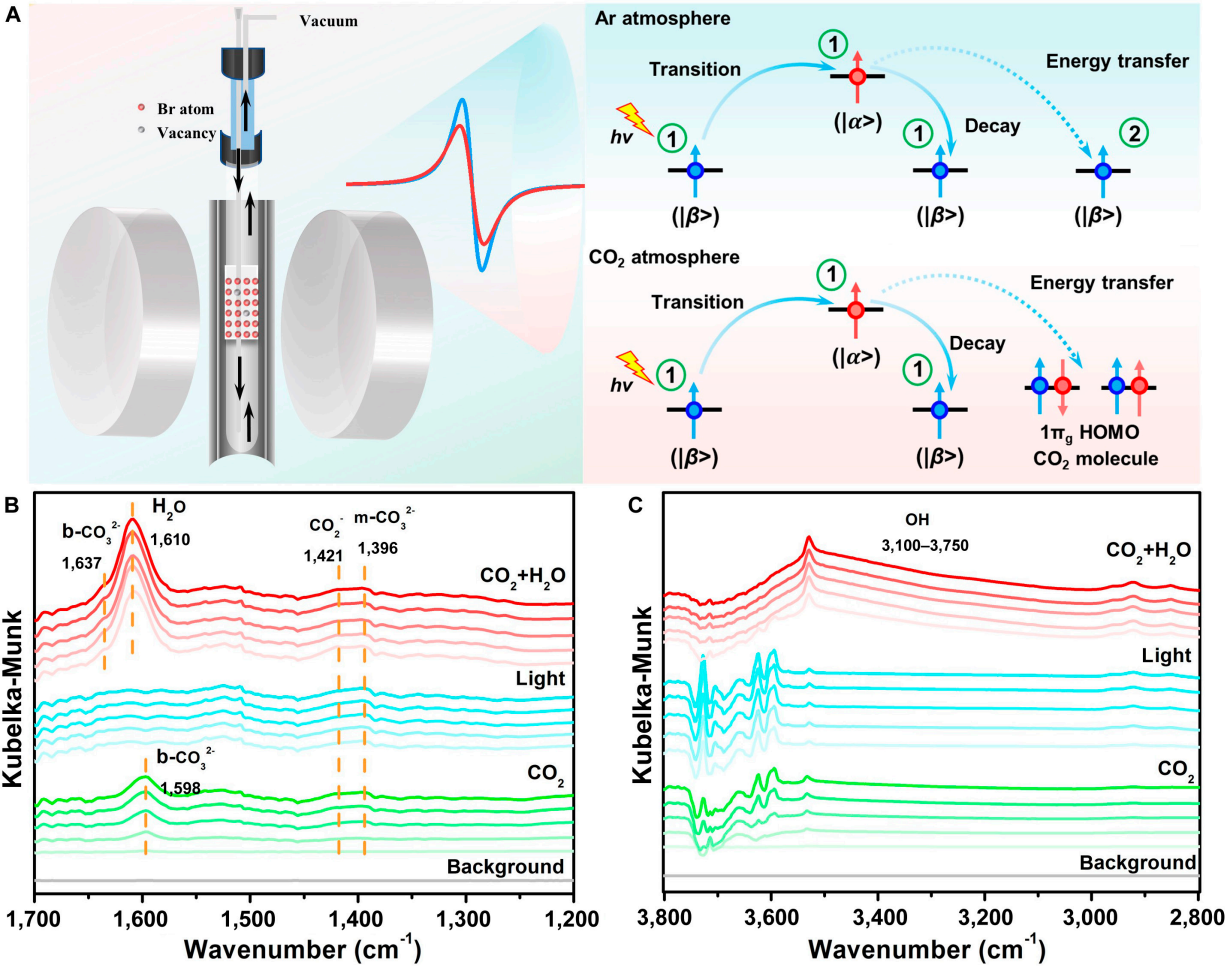
(A) The energy transfer process under different atmospheres. (B and C) In situ DRIFTS tests for CO_2_ interaction with BOB sample.

In addition, the pulsed EPR measurement of the SSR time T_2e_ was performed on the X-band EPR spectrometer (CIQTEK, EPR-100) with a dry cooling system. The T_2_ relaxation time before and after lighting in different atmospheres (Fig. 2D and Table S1) was compared. In the CO_2_ atmosphere, the T_2_ relaxation time after light irradiation is reduced by 198 ns compared with that in the dark condition, while it is only reduced by 23 ns in the Ar atmosphere. This result further confirms that CO_2_ molecules can lead to the reduction of T_2_ relaxation time. Of note, T_2_ relaxation time represents the recovery process of the lateral component, which is called lateral relaxation time, also known as SSR time. This process is completed through the energy relay between spin centers as shown in Fig. 3A. Under the Ar atmosphere, a low-energy electron (|*β*> state) could absorb microwave energy *hν* and transition to a high-energy state (|*α*> state), which could transfer the energy to the next low-energy electron and then itself returns to the initial low-energy state (|*β*> state). Under a CO_2_ atmosphere, the excited high-energy electron (|*α*> state) transfers energy to the CO_2_ molecule adsorbed on the surface; high-energy CO_2_ molecules are more likely to be activated. The result is further verified by density functional theory calculation; the CO_2_ molecule adsorbed on the Br vacancy displays more negative adsorption energy and more pronounced bond angle alteration than that on the BOB sample without Br vacancy, which indicates higher thermodynamics feasibility. Consequently, the EPR linewidth broadens and the relaxation time decreases due to the strong interaction between the spin center and CO_2_ molecules, which is ascribed to the in situ generated surface Br vacancies. Subsequently, most of the CO_2_ molecules were desorbed from the surface and the EPR signal recovered after vacuum treatment of the sample. Very few CO_2_ molecules are strongly adsorbed or undergo chemical reactions, resulting in a small number of single electrons being consumed (Fig. S6). To further explore the interaction between CO_2_ molecules and defective surface, the formation of reaction intermediates is monitored by in situ DRIFTS technology in Fig. 3B and C. When only CO_2_ molecules flowed into the reactor, the IR peak at 1,421 cm^−1^ corresponding to ·${\text{CO}}_{2^{-}}$ can be observed, indicating that adsorbed CO_2_ molecules acquire electrons from the PI-BrVs. Additionally, the peak at 1,598 cm^−1^ and 1,396 cm^−1^ can be found, which could have contributed to the bidentate carbonate $\left(b-{\text{CO}}_{3^{2-}}\right)$ group and monodentate carbonate $\left(m-{\text{CO}}_{3^{2-}}\right)$ group, respectively [39]. This carbonate most likely derived from the interaction between CO_2_ and residual water vapor in the reactor, while the peak at 1,598 cm^−1^ disappeared under light irradiation, demonstrating that the dissociation of the bidentate carbonate occurs in the absence of water vapor. Subsequently, both CO_2_ molecules and water vapor were simultaneously introduced into the reaction chamber. In addition, a new IR peak at 1,637 cm^−1^ was detected, which could be attributed to the COOH* species, considered as the critical active intermediate during photocatalytic CO_2_ reduction [40]. Meanwhile, the distinct peaks at 1,608 cm^−1^ and 3,100 to 3,750 cm^−1^ can be detected, which could be assigned to the adsorption of H_2_O molecules [34]. This result further evidences that the in situ generated PI-BrVs act as active sites to promote the adsorption and activation of CO_2_ molecules and H_2_O molecules.

### Exploring the effects of photoinduced Br vacancies on H_2_O molecules

**Fig. 4.**
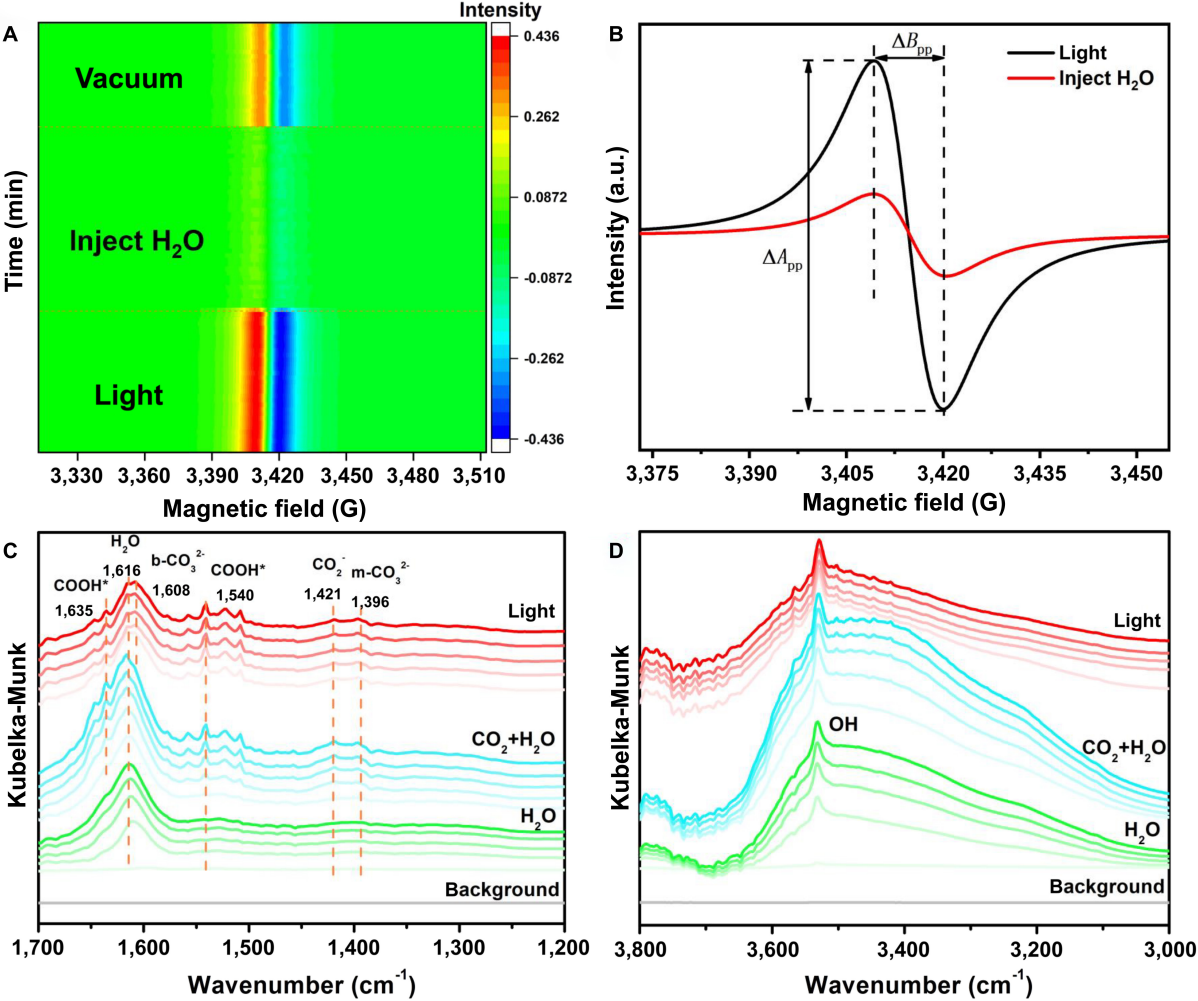
(A) In situ EPR visualization map of BOB sample under light irradiation, in H_2_O vapor atmosphere, and in a vacuum, respectively. (B) EPR spectra before and after the injection of H_2_O vapor. (C and D) In situ DRIFTS tests of the interaction between H_2_O molecules and BOB sample under light irradiation.

Reactant molecule adsorption and activation are an essential step in catalytic reaction, with the H_2_O molecule playing a critical role in photocatalytic CO_2_ reduction. In general, H_2_O molecules can provide a proton source for the CO_2_ hydrogenation process. To explore the adsorption and activation of H_2_O molecules on the PI-BrVs, an in situ EPR experiment in a moist Ar atmosphere (H_2_O/Ar) was carried out. As shown in Fig. 4A, after the generation of PI-BrVs under light irradiation, the EPR signal decreases with the injection of water vapor, indicating that H_2_O molecules adsorb on the PI-BrVs and interact with surface single electrons. Subsequently, the EPR signal is partially restored with the removal of H_2_O vapor in a vacuum (Fig. S7). This phenomenon demonstrates that, in a vacuum, the weakly adsorbed H_2_O molecules desorbed, while the strongly adsorbed or chemically reacted H_2_O molecules consumed electrons, leading to the part of the restoration of the EPR signal. Therefore, the H_2_O molecule could be adsorbed and activated by electron transfer on PI-BrV sites. The H_2_O molecule, as a proton donor during the reduction process of CO_2_, consumes electrons to generate the H* and OH^−^.

$$H_{2}O+e^{-}\to H^{*}+{\mathrm{OH}}^{-}$$ (5)

Importantly, there is no significant change in the linewidth of the EPR spectrum after the injection of H_2_O molecules (Fig. 4B), which was different from the phenomenon of direct injection of CO_2_ molecules, further indicating the special energy exchange between CO_2_ molecules and the surface of PI-BrVs. To study H_2_O molecules’ adsorption and activation on the defect surface, in situ DRIFTS was performed in Fig. 4C and D. Upon introducing H_2_O molecules into the reaction chamber, an obvious IR peak at 1,616 cm^−1^ emerged, which could be attributed to the deformation vibrations of adsorbed H_2_O molecules. In the meantime, the stretching vibrations of hydroxyl groups (3,050 to 3,700 cm^−1^) were observed and the peak strength evidently increased with the accumulation of adsorption time. These results suggest that the generation of Br vacancies greatly enhanced the adsorption of H_2_O molecules, which lays the foundation for further photocatalytic CO_2_ reduction reactions. Subsequently, new peaks at 1,608 cm^−1^, 1,421 cm^−1^, and 1,396 cm^−1^ could be found when CO_2_ molecules and H_2_O molecules were injected into the reaction chamber together, which could be ascribed to $b-{\text{CO}}_{3^{2-}}$, ·${\text{CO}}_{2^{-}}$, and $m-{\text{CO}}_{3^{2-}}$. More importantly, peaks at 1,635 cm^−1^ and 1,540 cm^−1^ were observed and corresponded to COOH*, an essential intermediate in CO_2_ reduction to CO. After the light irradiation, the intensity of the peaks (at 1,616 cm^−1^ and 3,050 to 3,700 cm^−1^) associated with H_2_O molecules decreased dramatically, further evidencing the activation and consumption of H_2_O molecules, which could provide efficient proton for further photocatalytic CO_2_ reduction reaction.

### Investigating the impact of photoinduced Br vacancies on photocatalytic CO_2_ reduction

**Fig. 5.**
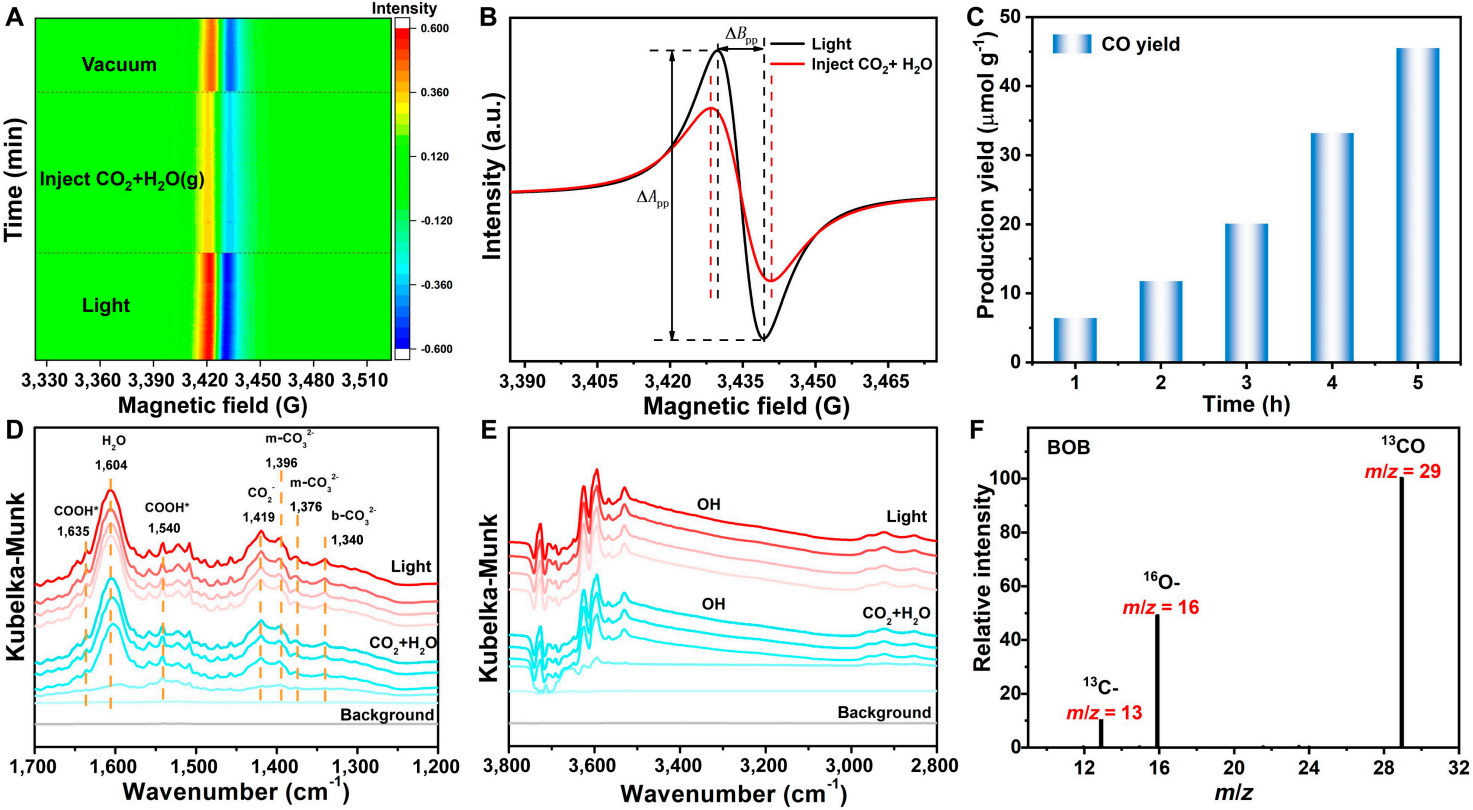
(A) In situ EPR visualization map of BOB sample under light irradiation, in a moist CO_2_ atmosphere, and in a vacuum. (B) EPR spectra before and after moist CO_2_ gas injection. (C) Photocatalytic CO_2_ reduction reaction performance of BOB sample. (D and E) In situ DRIFTS tests for moist CO_2_ molecules interaction with BOB sample under light irradiation. (F) The ^13^CO_2_ isotopic labeling experiment during photocatalytic reduction of ^13^CO_2_.

In the above experiments, the adsorption and activation of CO_2_ and H_2_O molecules on the PI-BrVs were investigated. To unravel the inherent reason for the CO_2_ photoreduction properties, in situ EPR experiments in a mixture atmosphere (CO_2_/H_2_O) were further performed to explore the real reaction mechanism by simulating an actual photocatalytic environment. When the reactant molecules (CO_2_/H_2_O) were introduced into the reactor, the EPR signal reduction is attributed to the single-electron transfer from PI-BrVs to CO_2_ and H_2_O molecules (Fig. 5A). The result indicates that the strong electronic interaction between Br vacancies and reactant molecules could facilitate CO_2_ and H_2_O molecules’ adsorption and activation. Subsequently, when the reactor is in a vacuum, some of the weakly adsorbed reactant molecules are desorbed from the defect sites, while the strongly adsorbed or chemically reactive molecules cannot desorb from the catalytic surface, resulting in the recovery of partial single-electron signals (Fig. S8). More importantly, the EPR signal line width broadened with the injection of the mixture (CO_2_ and H_2_O), resulting in a shortened relaxation time. The shorter the relaxation time, the more efficient the energy transfer between spin and environmental, indicating that the reactant molecules interact more strongly with the PI-BrVs. In situ DRIFTS was used to probe reaction intermediates and elucidate the potential reaction pathways (Fig. 5D and E). A new peak at 1,419 cm^−1^ was observed, corresponding closely to the carboxylate (·${\text{CO}}_{2^{-}}$) vibration frequency. Furthermore, CO_2_ molecules and H_2_O molecules co-adsorbed on the Br vacancies, leading to the creation of the monodentate carbonate $\left(m-{\text{CO}}_{3^{2-}}\right)$ group at 1,396 cm^−1^ and the asymmetric OCO stretches of the bidentate carbonate $\left(b-{\text{CO}}_{3^{2-}}\right)$ group at 1,340 cm^−1^ [41]. More importantly, the COOH* is regarded as one of the most crucial intermediates for converting CO_2_ to CO, and its related peaks at 1,635 cm^−1^ and 1,540 cm^−1^ could be found [36,42]. The most likely reduction pathway for photocatalytic CO_2_ reduction is proposed as follows based on the above in situ DRIFTS and in situ EPR experiments:

$$\begin{array}{l} H_{2}O+e\to H^{*}+{\mathrm{OH}}^{-}\\ {\mathrm{CO}}_{2}+*\to {\mathrm{CO}}_{2}^{*}\\ {\mathrm{CO}}_{2}^{*}+H^{*}\to \mathrm{CO}+H_{2}O\\ {\text{COOH}}^{*}+H^{*}\to \mathrm{CO}+H_{2}O \end{array}$$ (6)

During the process of photoreduction of CO_2_, the BOB sample could produce 45.51 μmol g^−1^ of CO after 5 h of the photoreduction reaction (Fig. 5C). To assess its stability, the BOB sample was tested for an extended period with 10 h of light exposure. Figure S9 shows the linear growth of CO yields with increasing illumination time, providing strong evidence of good stability. In addition, the isotope probing method is employed to trace the source of C, where ^13^CO_2_ is used as the carbon feedstock (Fig. 5F). A peak at *m*/*z* = 29 was detected by mass spectroscopy, which was ascribed to the ^13^CO. This is powerful evidence for CO generation through light-induced CO_2_. In addition, the absence of a ^13^CH_4_ peak in the isotopic labeling test is consistent with the absence of CH_4_ or H_2_ detected in Figs. S10 and S11. This result suggests 100% catalytic selectivity of stable photocatalytic CO_2_ reduction reaction on the BOB sample. Compared with previously reported bismuth-based photocatalysts, BOB with PI-BrVs has a higher CO generation rate and product selectivity (Table S2). Overall, the interaction between PI-BrVs and reactant molecules promotes the photocatalytic CO_2_ reduction reaction.

## Discussion

In this work, using the tetragonal BOB as a prototype, we reveal the dynamic behavior of active sites and elucidate the process of energy exchange between PI-BrVs and CO_2_ molecules. In situ cw-EPR and pulsed EPR technologies reveal that the SSR time (T_2_) of BOB is decreased by 198 ns during the photocatalytic CO_2_ reduction reaction, which is also confirmed by the broadened EPR linewidth. This result demonstrates that the excited spin center of PI-BrV can transfer energy to the ground-state CO_2_ molecules. The high-energy CO_2_ molecules are more likely to be activated and proceed to the subsequent CO_2_ reduction. In addition, theoretical calculations indicate that the bended CO_2_ adsorption configuration on the surface of BOB with Br vacancies caused the decrease of the LUMO of the CO_2_ molecule, which makes it easier for CO_2_ molecules to acquire electrons and get activated. The activated CO_2_ molecules can be converted into important COOH* intermediates, which would transform into the final product CO with a generation rate of 9.1 μmol g^−1^ h^−1^ and a selectivity of 100%. This work provides a new perspective for the activation mechanism of CO_2_ molecules on photoinduced active sites.

## Materials and Methods

### Reagents

All chemicals used were of analytical purity and required no additional treatment.

### Catalyst preparation

The BiOBr sample was prepared by a method of precipitation. Details of the experiment are shown in the Supplementary Materials.

###  In situ EPR test

The self-made in situ EPR reaction chamber is a quartz sample tube with an inner diameter of 8 mm. The type of EPR spectrometer is EMX nano (Bruker). Details of the experiment are shown in the Supplementary Materials.

### Evaluation of photoinduced Br^−^ migration

Photocatalyst (20 mg) was used in this test. The light source is a 300-W xenon lamp (PLS-SXE300UV, Beijing Perfectlight). The content of Br^−^ was detected by an IC (Shimadzu Essentia LC-16i chromatograph). Details of the experiment are shown in the Supplementary Materials.

###  In situ DRIFTS

 In situ DRIFTS test was implemented using a TENSOR II FT-IR spectrometer (Bruker) equipped with an in situ diffuse-reflectance chamber (Harrick) and a high-temperature reaction chamber (HVC). Further experiment details are available in the Supplementary Materials.

## Data Availability

All data needed for this study are available in the article and its Supplementary Materials files.
