# Supplementary material for: Rapid Energy Exchange between In Situ Formed Bromine Vacancies and CO2 Molecules Enhances CO2 Photoreduction
Source: Research (Wash D C). 2023 Oct 5;6:0244. doi: 10.34133/research.0244 (PMC10557117; doi:10.34133/research.0244)
Supplement: Supplementary 1 — Figs. S1 to S11 Tables S1 and S2 [file research.0244.f1.docx]

Supporting Information

**Rapid Energy Exchange between *in situ* Bromine Vacancies and CO_2_ molecules Facilitates Photocatalytic CO_2_ Reduction**

Qin Ren,^1^ Ye He,^2^ Hong Wang,^1^ Yanjuan Sun,^2^ Fan Dong ^1,^*

^1^ Research Center for Environmental and Energy Catalysis, Institute of Fundamental and Frontier Sciences, University of Electronic Science and Technology of China, Chengdu 611731, China.

^2^ School of Resources and Environment, University of Electronic Science and Technology of China, Chengdu 611731, China.

*E-mail: dfctbu@126.com, [dongfan@uestc.edu.cn](mailto:dongfan@uestc.edu.cn)

**Catalyst preparation**

4 mmol Bi(NO_3_)_3_•5H_2_O were dissolved in 30 mL ethylene glycol and 20 mL deionized water, then vigorous stirring for 30 minutes at room temperature. Next, 4 mmol KBr was dissolved in 10 mL deionized water. Subsequently, KBr solution was added to the solution above mentioned. The mixture was stirred for 2 h at room temperature. Finally, the precipitates washed with ethanol and distilled water, and then dried at 60° C in a vacuum and the sample were denoted as BOB.

**Characterizations**

Phase structures of the samples were analyzed by Shimadzu X-ray Diffractometer (XRD-6100, Japan) with Cu Kα radiation. Transmission electron microscopy (TEM; with an FEI Tecnai G2 F20 S-TWIN instrument; 200 kV, American). The surface properties were detected using X-ray photoelectron spectroscopy (XPS) with AlK^α^ X-rays (Thermo ESCALAB 250; Thermo Scientific, Waltham, MA, USA). The electron paramagnetic resonance (EPR) characterization (EMX nano, Bruker) of the as-prepared photocatalysts to detect oxygen vacancy at room temperature. The in situ FTIR spectroscopy (INVENIO R). The Raman spectroscopy (RENISHAW).

**Evaluation of photocatalytic activity**

The CO_2_ photoreduction performances were carried out in an intermittent flow system. A certain amount of photocatalyst was dispersed into deionized water under ultrasonication. Then 10.0 mg photocatalyst was loaded on the surface of a microporous membrane with a radius of 2.0 cm by filtration. The supported photocatalyst was placed on a tray in the photocatalytic reactor. After sealing, the reactor was vacuumed with a glass valve opened to exhaust the air in the reactor, and then high-purity CO_2_ (99.99 %) was introduced into the reactor. After repeating this 3 times, 0.15 mL of water was added to the reactor, and the valve was closed. The pressure in the reactor was typically regulated to 94-95 kPa, slightly lower than the atmospheric pressure, which helps maintain the reactor’s air tightness. Finally, the whole system was irradiated by simulated sunlight provided by a 300 W Xenon lamp coupled with an AM 1.5G filter. The photocatalytic reactor was connected to the gas chromatograph through an MC-SPB10 system (Beijing Merry Change Technology CO., LTD) during the irradiation, and the gas was collected every hour.

**Number of Spins Calculation**

The Spin Calculation procedure can acquire the number of unpaired spins in the sample from the EPR spectrum without the additional steps of preparing and measuring a standard. Equation 1 shows the parameters used for the determination of the number of spins in a sample. The acquisition parameters are stored together with the experimental spectrum along with the resonator properties which are set by a factory calibration of the resonator with a standard of known concentration. The double integral is determined from a normalized EPR signal, making the signal intensity independent of receiver gain, conversion time, and the number of scans. The sample temperature is constant and known). The Boltzmann factor is calculated from the microwave frequency and sample temperature (a default temperature of 298 K) stored with the EPR spectrum.

Equation 1: $N_{S}= \frac{DI V}{P_{1/{2c}}B_{m} Q c S\left( S+1 \right) n_{B}f(B_{1}{,B}_{m})}$

DI: Double Integral, V: Sample Volume, P: Microwave Power, $B_{m}$: Modulation Amplitude

Q: Resonator Q-factor, c: Resonator Calibration Factor, S: Electron Spin, $n_{B}$: Boltzmann Factor, f($B_{1}, B_{m}$): Resonator Field Profile.

***In situ* EPR test**

For sample preparation, 100 mg of catalyst was placed in an 8 mm inner diameter quartz sample tube for *in situ* EPR tests. Samples were *in situ* characterized at room temperature under different atmospheres (CO_2_, H_2_O) by using a continuous wave EPR spectrometer (Bruker, EMX nano), operating at X-ban frequencies (9.6 GHz). The signal was collected every 20 s (note that it takes 40 s to san an EPR spectrum). The xenon lamp (LSN 155, Germany) was used as the light source. The relevant test parameters are as follows: Modulation Amplitude of 2 G, Microwave Power of 1 mW, Conversion Time of 40 ms, and Time Constant of 40 ms.

**DFT calculations**

All calculations have been performed with the Vienna Ab-initio Simulation Package (VASP5.4.1). The generalized gradient approximation with Perdew-Burke-Ernzerhof (PBE) exchange and correlation functional was utilized. A 3×3×1 K-point was sampled in the Brillouin zone. A plane wave energy cutoff of 400 eV was used to optimize the geometric structures, and the energy and electronic forces were converged to 1×10^-4^ eV and 0.03 eV/Å, respectively.

***In situ* FT-IR**

*In situ* FT-IR test was performed with a TENSOR II FT-IR spectrometer (Bruker) equipped with an *in situ* diffuse-reflectance chamber (Harrick) and a high-temperature reaction chamber (HVC). The reactor contained three windows, including two ZnSe windows for IR measurements and a quartz window for light irradiation using a Xe lamp (MVL-210, Japan). The samples were pretreated inside the chamber in Ar at 100 °C for 30 min to clean the surface of samples. The background spectrum was then collected after the chamber temperature was lowered to room temperature. Next, the reaction gas (CO_2_ 20 mL min^−1^, H_2_O/Ar 10 mL min^−1^) was introduced into the reactor, and the variation of the FTIR spectra were recorded to monitor the dynamic adsorption process. After reaching sorption equilibrium (30 min), the background spectrum was collected again. Subsequently, the light source (300 W xenon lamp, AM1.5 filter) was turned on, and the FTIR spectra were recorded as a function of time to investigate the dynamics of the reduction of the reactants under irradiation.

**Quasi*-in situ* XPS**

Quasi-*in-situ* XPS was carried out with monochromatic Al K_α_ (1486.6 eV) radiation. The instrument model was ESCALAB Xi^+^ electron spectrometer (Thermo Scientific, USA). For quasi-*in-situ* XPS, the spectra were acquired after UV irradiation 15 min of UV irradiation.

**Supplementary Figures**

**
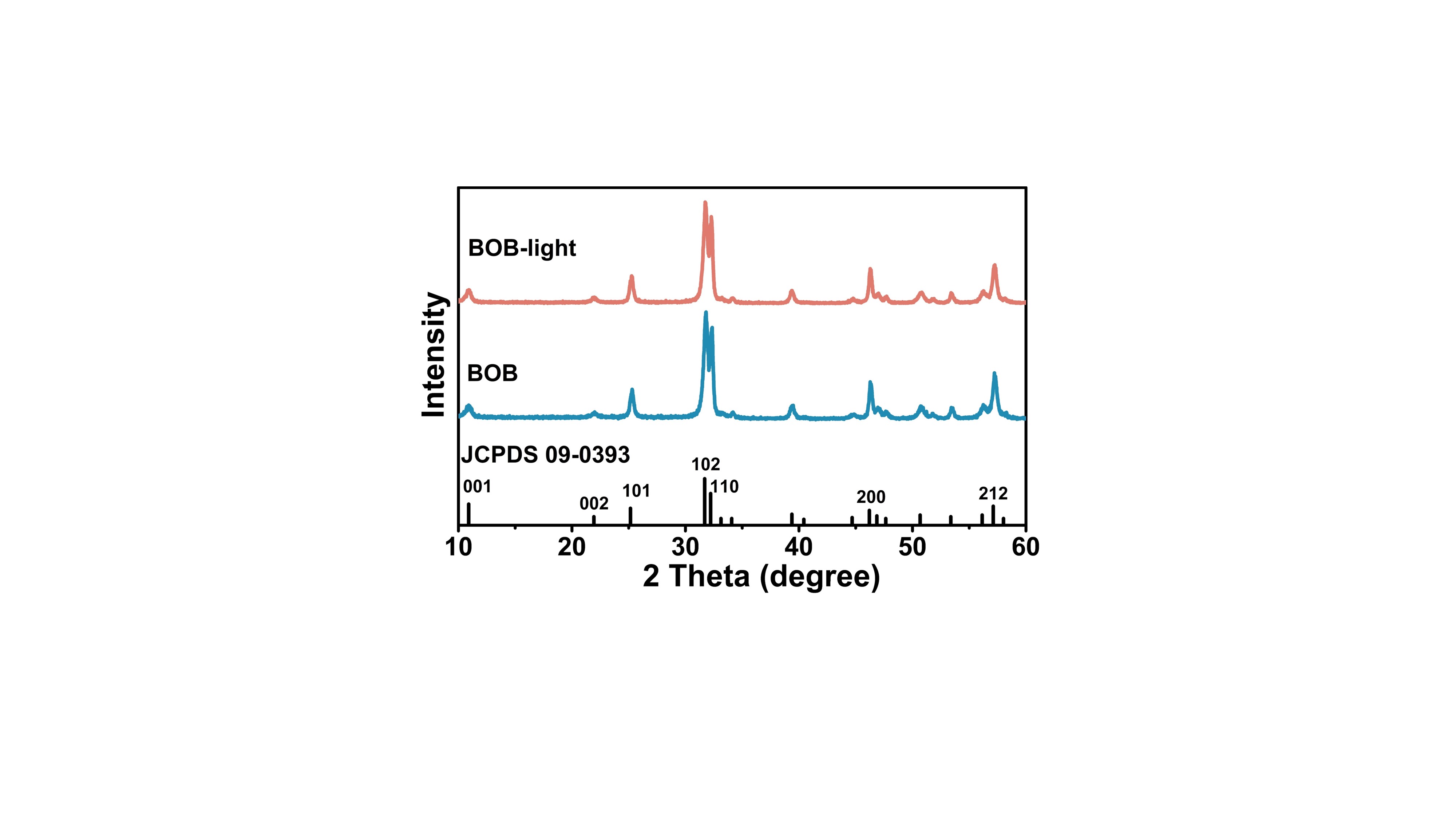
**

**Figure. S1.** XRD of BOB samples.

**
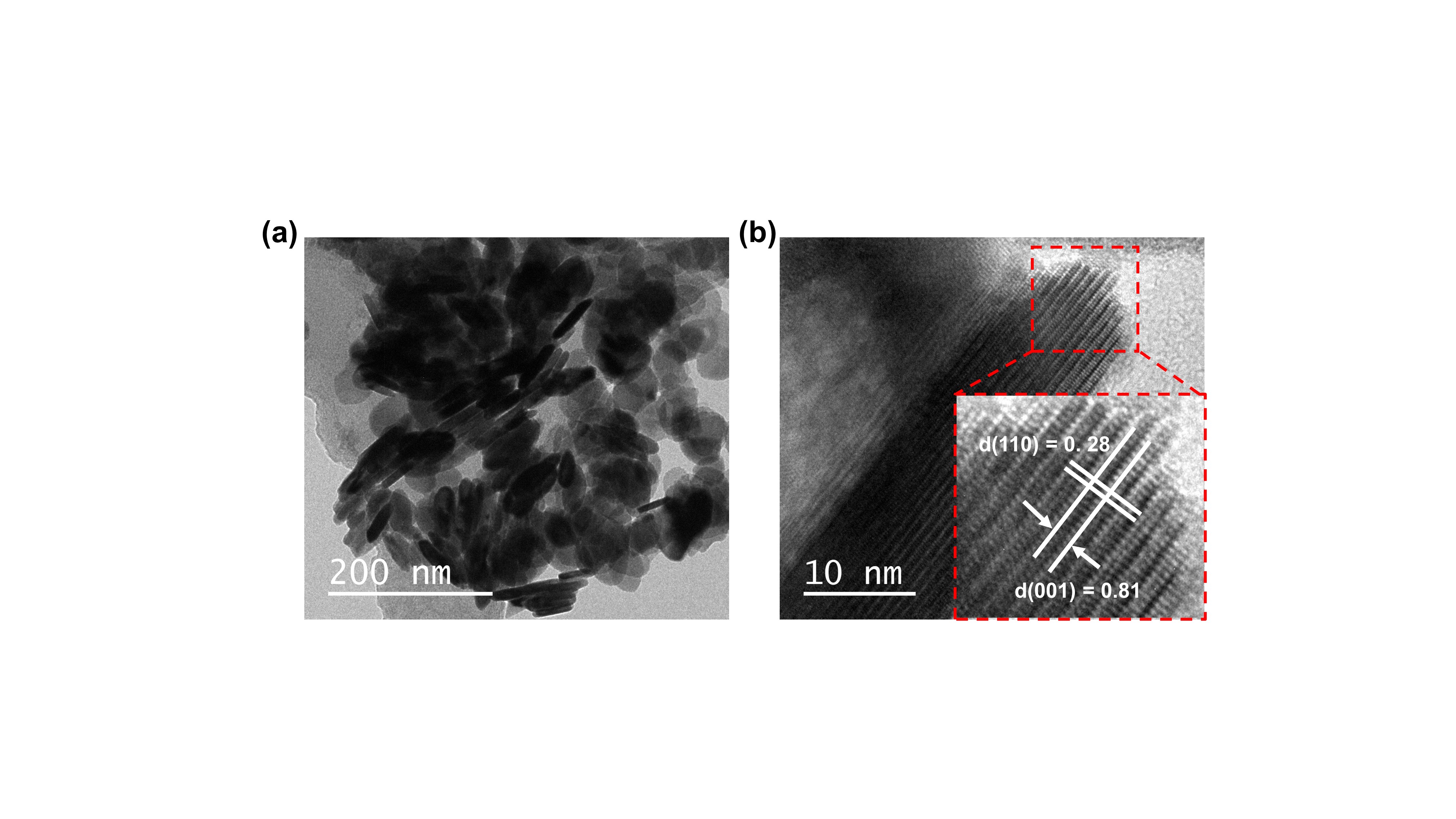
**

**Figure. S2.** **(a)** TEM image of BOB samples. **(b)** HRTEM image of BOB samples.


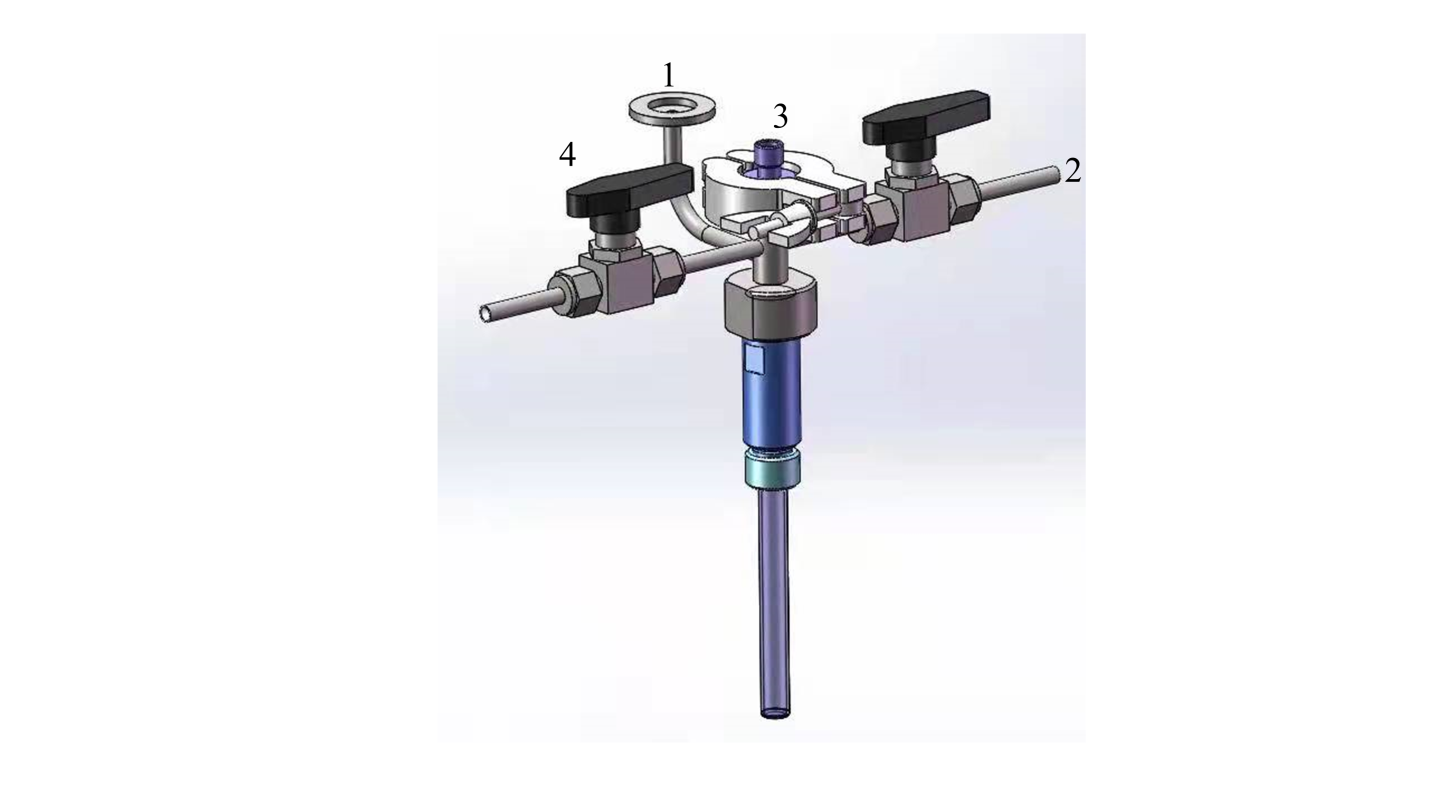


**Figure. S3.** The photograph of the *in situ* EPR reactor. 1: Connect to the vacuum gauge; 2: connect to the mechanical pump and molecular pump set. 3: Inject gas into the reactor. 4: in the closed state.

**
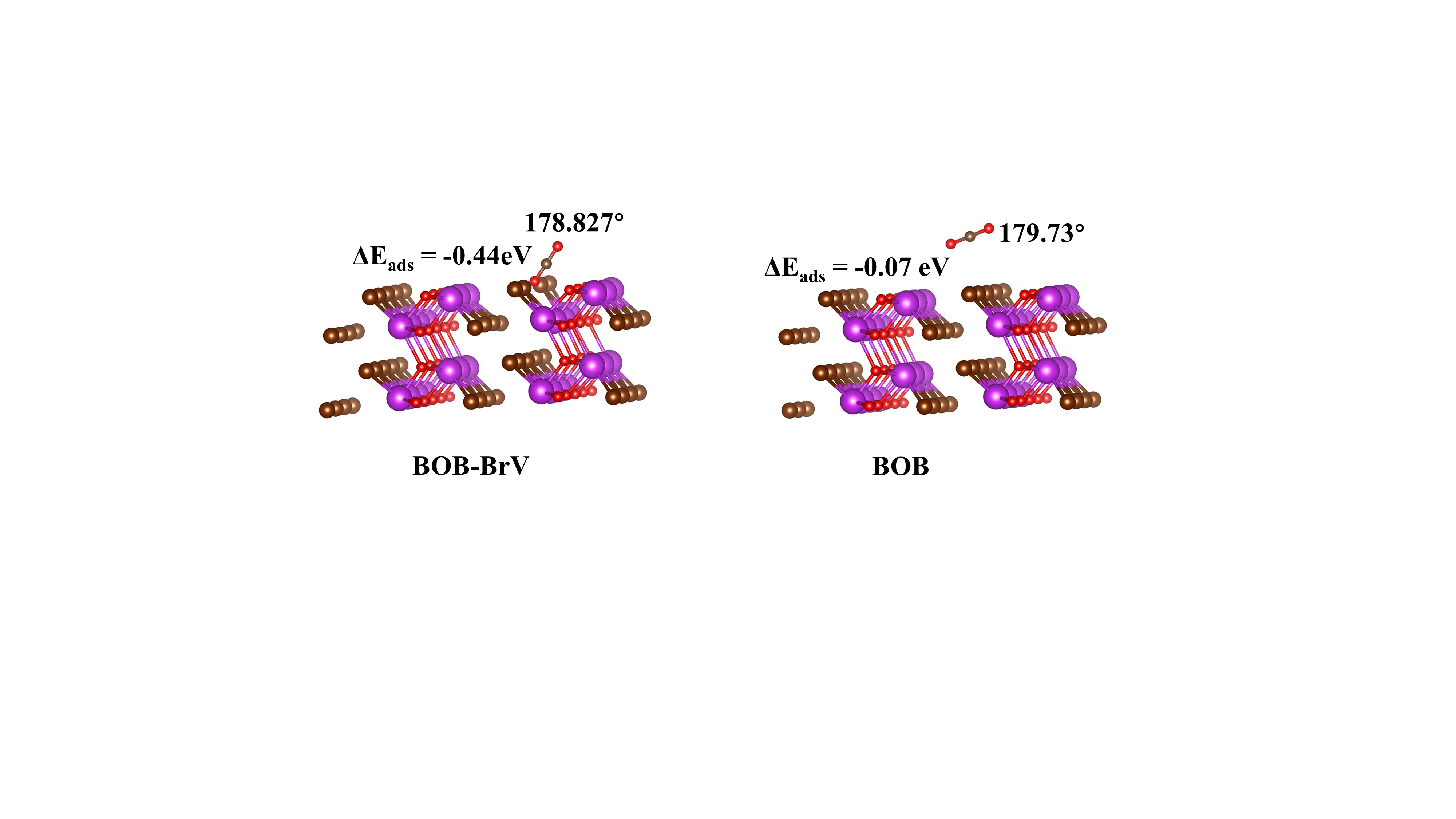
**

**Figure. S4. (a)** The adsorption energy of CO_2_ molecuels on BOB-BrV and **(b)**BOB.


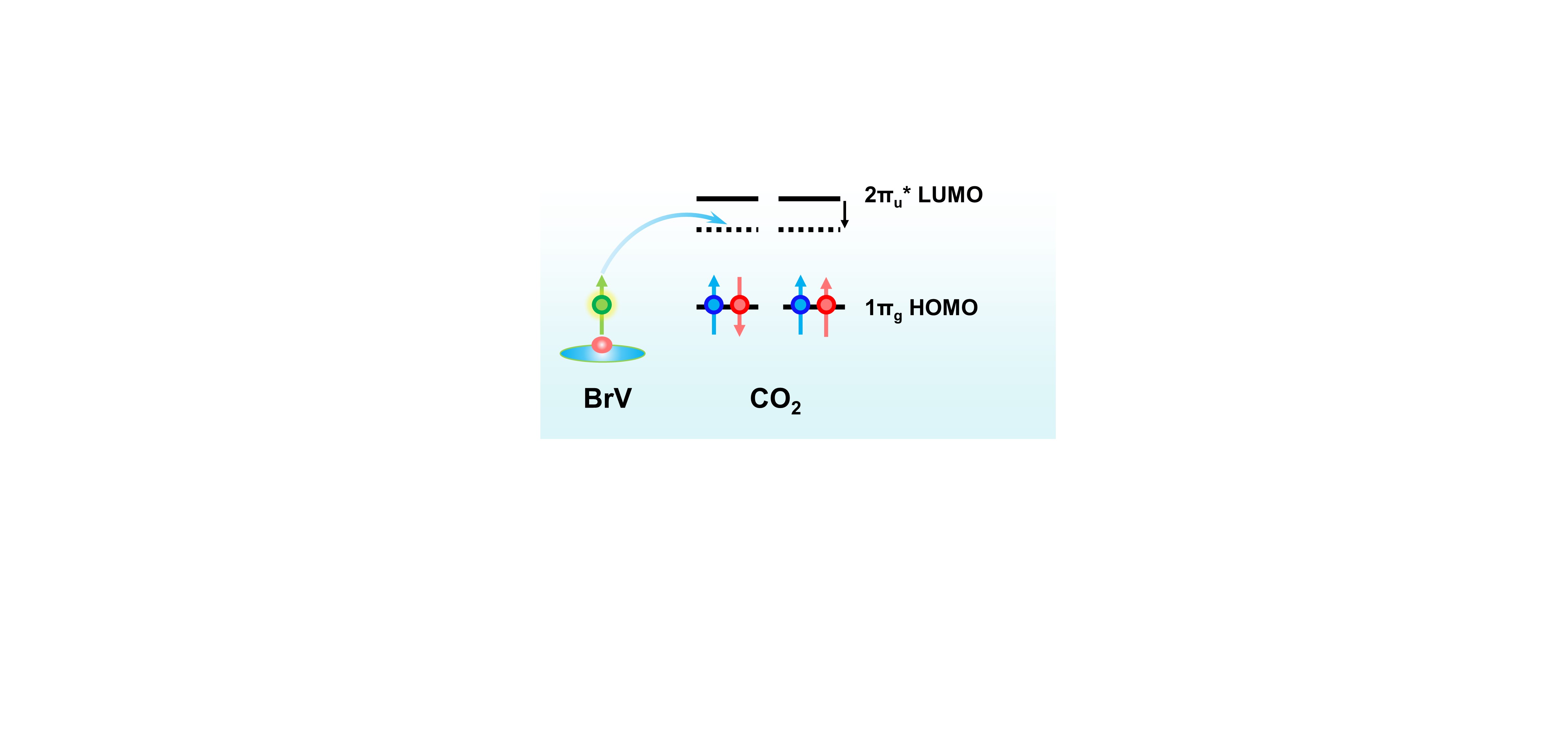


**Fig. S5.** The electron transfer between bromine vacancy and CO_2_ molecules.


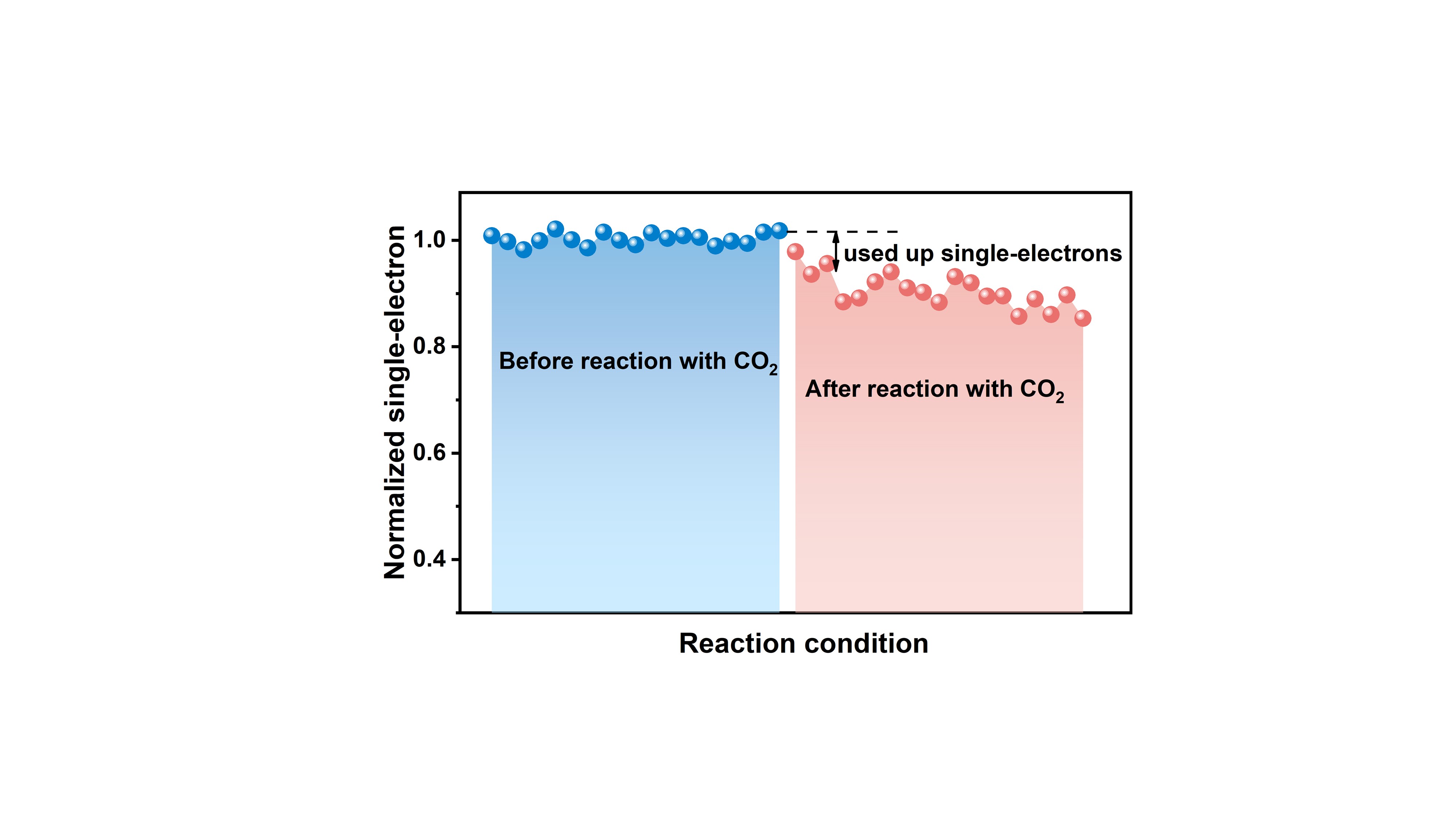


**Fig. S6.** The number of single-electron about before and after reaction with CO_2_ molecules.


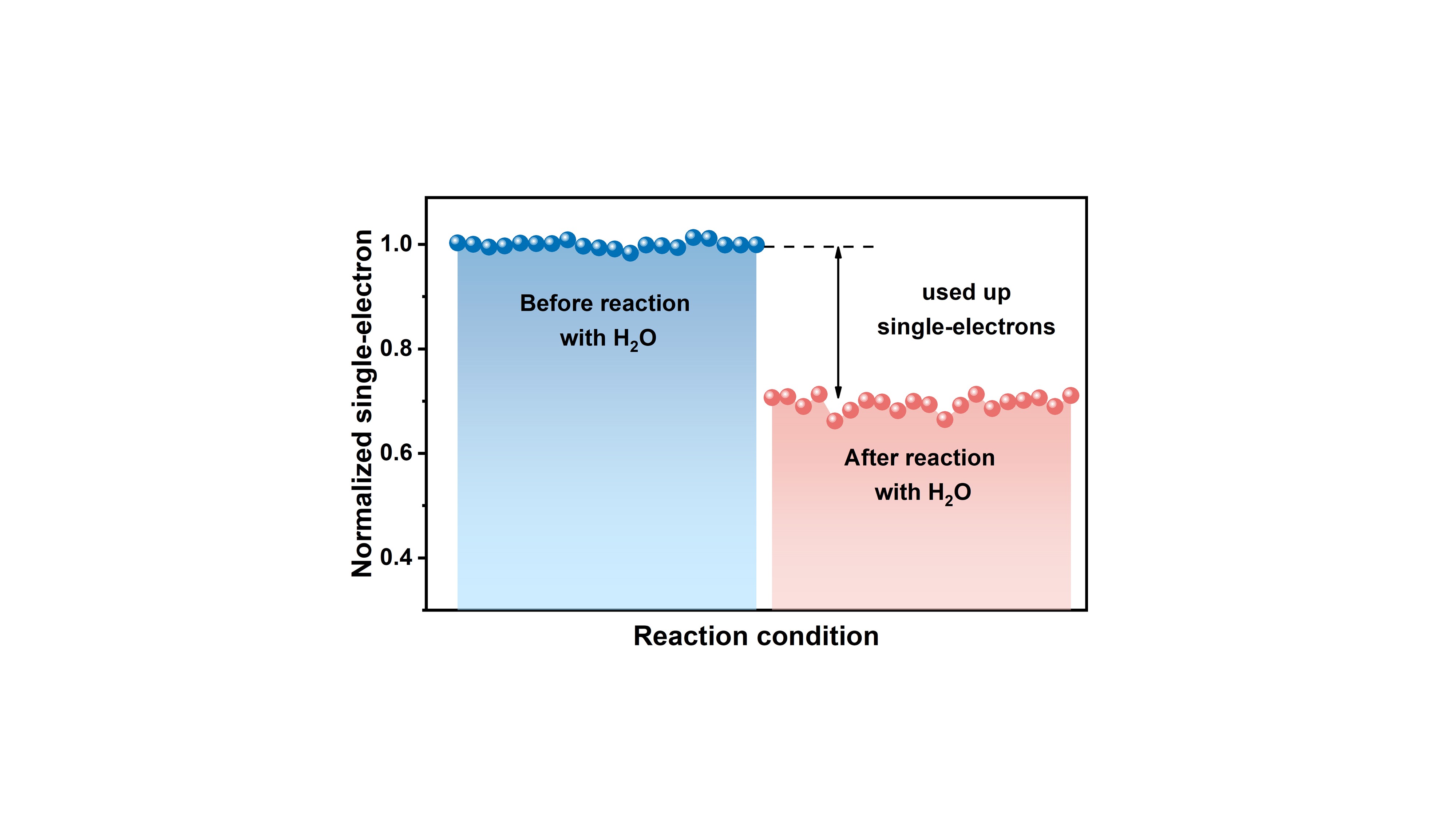


**Fig. S7.** The number of single-electron about before and after reaction with H_2_O molecules.

**
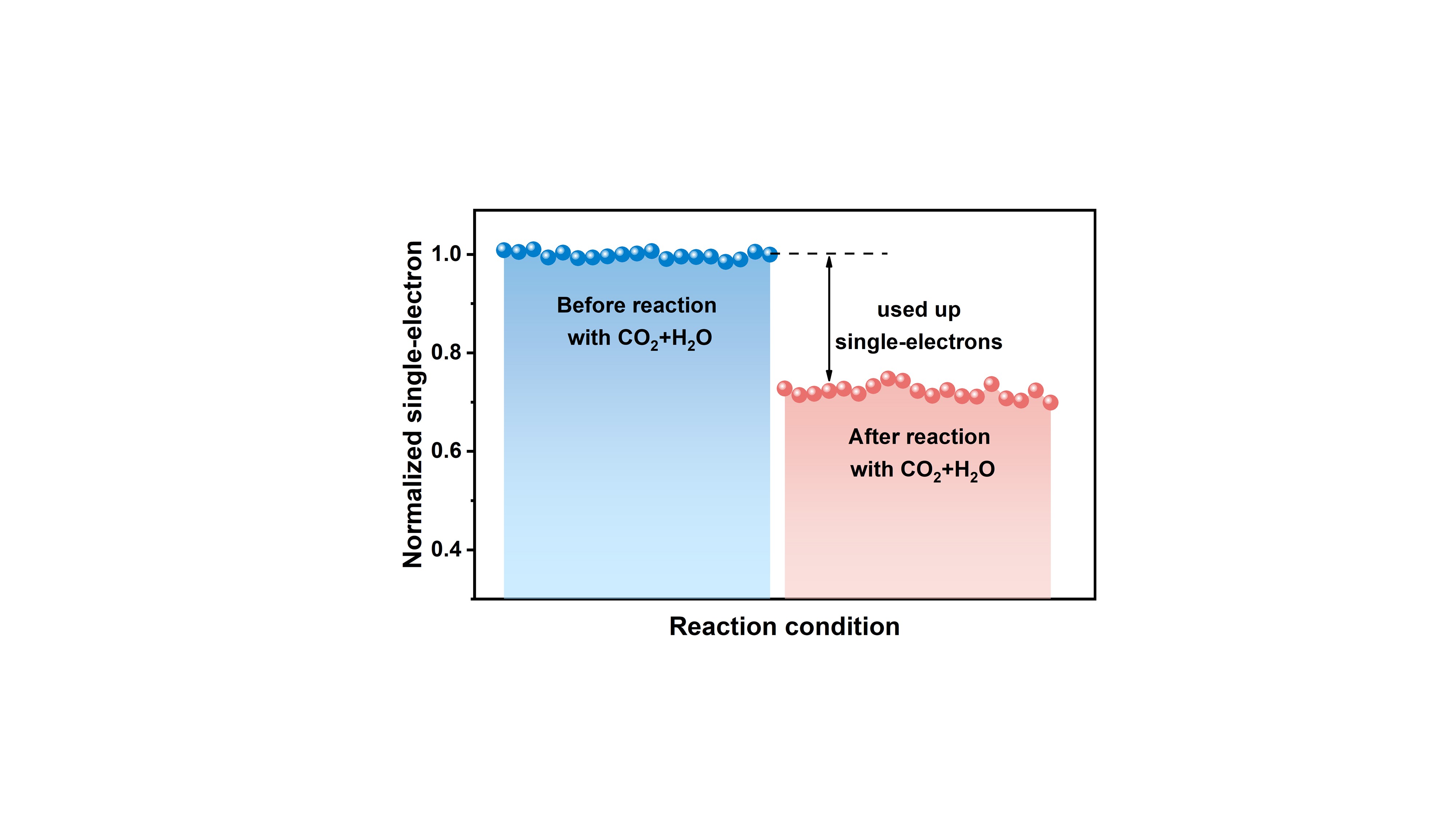
**

**Fig. S8.** The number of single-electron about before and after reaction with CO_2_ and H_2_O molecules.

**
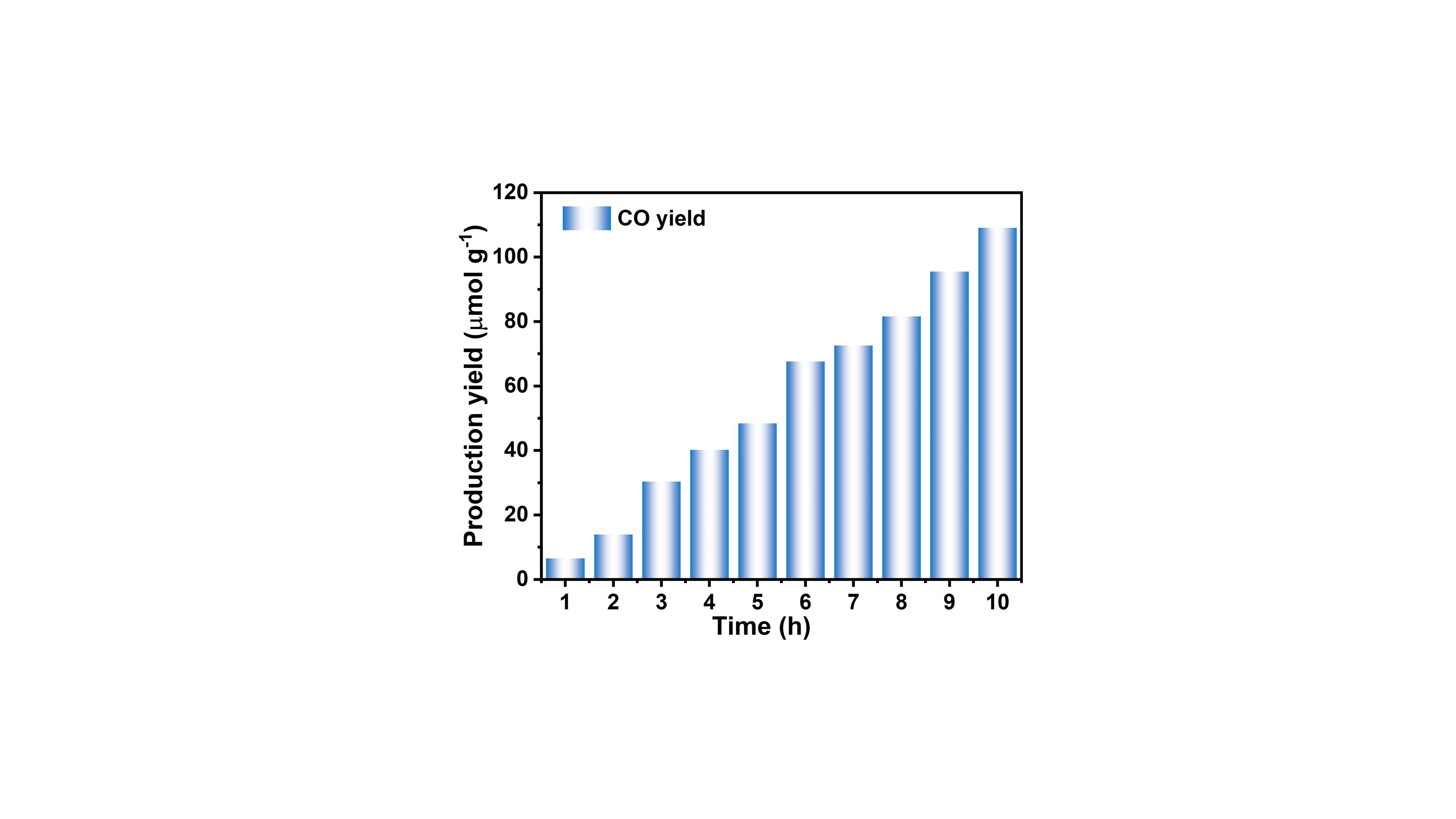
**

**Fig. S9.** The stability of BOB samples during photocatalytic CO_2_ reduction reaction.





**Fig. S10.** CH_4_ yield during CO_2_ photoreduction.





**Fig. S11.** H_2_ yield during CO_2_ photoreduction.

Supplementary Tables

**Supplementary Table S1.** T_2_ relaxation time in different conditions

|  | in dark | light | ΔT_2_ |
| --- | --- | --- | --- |
| Ar | 3012 ns | 2989 ns | 23 ns |
| CO_2_ | 3270 ns | 3072 ns | 198 ns |

**Supplementary Table S1.** Comparasion of the CO_2_ photoreduction activity to yield CO with other catalysts

| Entry | Photocatalyst | Light source | CO production (μmol g^-1^ h^-1^) | Product selectivity (%) | Ref |
| --- | --- | --- | --- | --- | --- |
| 1 | Hollow Bi_4_O_5_Br_2_ | 300W Xe lamp | 3.16 | 61.2% | [[1](#_ENREF_1)] |
| 2 | BiOBr | 300W Xe lamp (λ > 400 nm) | 1.68 | 72.4% | [[2](#_ENREF_2)] |
| 3 | BiOI | 300 W Xe lamp (λ > 400 nm) | 0.51 | 41.5% | [[3](#_ENREF_3)] |
| 4 | Bi_5_O_7_I | 300 W Xe lamp (λ > 400 nm) | 1.73 | 70.6% | [[4](#_ENREF_4)] |
| 5 | g-C_3_N_4_/BiOI | 300 W Xe lamp (λ > 400 nm) | 4.86 | -- | [[5](#_ENREF_5)] |
| 6 | BiOCl | λ > 420 nm | 3.94 | -- | [[6](#_ENREF_6)] |
| 7 | BiOBr | λ > 420 nm | 2.15 | -- | [[7](#_ENREF_7)] |
| 9 | Bi_2_MoO_6_ | 300 W Xe lamp | 3.62 | -- | [[8](#_ENREF_8)] |
| 10 | BiOBr with PI-BrVs | 300 W Xe lamp (with AM1.5 filter) | 9.1 | 100% | **This work** |

Supplementary References

1. Jin, X.; Lv, C.; Zhou, X.; Xie, H.; Sun, S.; Liu, Y.; Meng, Q.; Chen, G., A bismuth rich hollow Bi_4_O_5_Br_2_ photocatalyst enables dramatic CO_2_ reduction activity. *Nano Energy* **2019**, *64*.

2. Ye, L.; Jin, X.; Liu, C.; Ding, C.; Xie, H.; Chu, K. H.; Wong, P. K., Thickness-ultrathin and bismuth-rich strategies for BiOBr to enhance photoreduction of CO_2_ into solar fuels. *Appl. Catal. B* **2016**, *187*, 281-290.

3. Ye, L.; Wang, H.; Jin, X.; Su, Y.; Wang, D.; Xie, H.; Liu, X.; Liu, X., Synthesis of olive-green few-layered BiOI for efficient photoreduction of CO_2_ into solar fuels under visible/near-infrared light. *Sol Energy Mater Sol. Cells* **2016**, *144*, 732-739.

4. Ding, C.; Ye, L.; Zhao, Q.; Zhong, Z.; Liu, K.; Xie, H.; Bao, K.; Zhang, X.; Huang, Z., Synthesis of Bi_x_O_y_I_z_ from molecular precursor and selective photoreduction of CO_2_ into CO. *J. CO_2_ Util.* **2016_,_** *14*, 135-142.

5. Wang, J. C.; Yao, H. C.; Fan, Z. Y.; Zhang, L.; Wang, J. S.; Zang, S. Q.; Li, Z. J., Indirect Z-Scheme BiOI/g-C_3_N_4_ Photocatalysts with Enhanced Photoreduction CO_2_ Activity under Visible Light Irradiation. *ACS Appl. Mater. Interfaces* **2016**, *8* (6), 3765-75.

6. Li, M.; Zhang, Y.; Li, X.; Wang, Y.; Dong, F.; Ye, L.; Yu, S.; Huang, H., Nature-Derived Approach to Oxygen and Chlorine Dual-Vacancies for Efficient Photocatalysis and Photoelectrochemistry. *ACS Sustain. Chem. Eng.* **2018**, *6* (2), 2395-2406.

7. Kong, X. Y.; Ng, B.-J.; Tan, K. H.; Chen, X.; Wang, H.; Mohamed, A. R.; Chai, S.-P., Simultaneous generation of oxygen vacancies on ultrathin BiOBr nanosheets during visible-light-driven CO_2_ photoreduction evoked superior activity and long-term stability. *Catal. Today* **2018**, *314*, 20-27.

8. Di, J.; Zhao, X.; Lian, C.; Ji, M.; Xia, J.; Xiong, J.; Zhou, W.; Cao, X.; She, Y.; Liu, H.; Loh, K. P.; Pennycook, S. J.; Li, H.; Liu, Z., Atomically-thin Bi_2_MoO_6_ nanosheets with vacancy pairs for improved photocatalytic CO_2_ reduction. *Nano Energy* **2019,** *61*, 54-59.
